# *Wolbachia* Has Two Different Localization Patterns in Whitefly *Bemisia tabaci* AsiaII7 Species

**DOI:** 10.1371/journal.pone.0162558

**Published:** 2016-09-09

**Authors:** Peiqiong Shi, Zhan He, Shaojian Li, Xuan An, Ning Lv, Murad Ghanim, Andrew G. S. Cuthbertson, Shun-Xiang Ren, Bao-Li Qiu

**Affiliations:** 1 Department of Entomology, South China Agricultural University, Guangzhou, China; 2 Key Laboratory of Bio-Pesticide Innovation and Application, Engineering Technology Research Center of Agricultural Pest Biocontrol of Guangdong Province, Guangzhou, China; 3 Department of Entomology, Agricultural Research Organization, The Volcani Center, Bet Dagan, Israel; 4 Fera, Sand Hutton, York, United Kingdom; Universite de Poitiers, FRANCE

## Abstract

The whitefly *Bemisia tabaci* is a cosmopolitan insect species complex that harbors the obligate primary symbiont *Portiera aleyrodidarum* and several facultative secondary symbionts including *Wolbachia*, which have diverse influences on the host biology. Here, for the first time, we revealed two different localization patterns of *Wolbachia* present in the immature and adult stages of *B*. *tabaci* AsiaII7 cryptic species. In the confined pattern, *Wolbachia* was restricted to the bacteriocytes, while in the scattered pattern *Wolbachia* localized in the bacteriocytes, haemolymph and other organs simultaneously. Our results further indicated that, the proportion of *B*. *tabaci* AsiaII7 individuals with scattered *Wolbachia* were significantly lower than that of confined *Wolbachia*, and the distribution patterns of *Wolbachia* were not associated with the developmental stage or sex of whitefly host. This study will provide a new insight into the various transmission routes of *Wolbachia* in different whitefly species.

## Introduction

The associations among inherited bacterial symbionts and arthropods are very common in nature [1, 2], and these symbionts can be defined as primary or secondary ones as per their biological effects on arthropod hosts. The primary symbionts (such as *Portiera* in whitefly and *Carsonella* in psyllid) are obligate and have mutualism relationships with their hosts, providing essential nutrients under limited or unbalanced diets. Primary symbionts are generally localized in specialized cells called bacteriocytes, grouped together in a bacteriome [3]. In some cases, the primary endosymbionts become part of the “extended genome” of their host, being transferred vertically from a female host to her progeny [4]. Secondary endosymbionts are usually not required for the survival or reproduction of their hosts, but they may manipulate host reproduction, or help in the host’s defense against thermal stress, natural enemies and pathogens [5–8]. Similar to the primary endosymbionts, secondary endosymbionts are usually present in the gonads of hosts and can be transmitted vertically [9]. However, sometimes they are also been found in haemolymph, malpighian tubules, salivary glands, fat body, ovarian cells, gut structures and brains of their hosts [10–14]. Among the secondary endosymbionts, *Wolbachia* is one of the most abundant species that infects insects, mites, spiders and isopods [9, 15–18]. It is well-established that *Wolbachia* can promote its own transmission throughout the host population by manipulating host reproduction. The typical manipulation includes cytoplasmic incompatibility (CI), parthenogenesis, feminization and male-killing [19].

Many arthropod individuals harbor more than one species of endosymbiont, and the possibilities of endosymbiont horizontal transmission may be highly associated with their localizations in hosts, which are known to be much diverse. To know the spatial distribution of endosymbionts in their hosts is the cornerstone in understanding the key aspects of symbiont-host interactions as well as the fitness, phenotype and dynamics of these bacteria. Caspi-Fluger *et al*. [3] found two distribution patterns of *Rickettsia* in the sweetpotato whitefly *Bemisia tabaci*, the scattered pattern located throughout the host hemocoel but not in the bacteriocytes, while another confined pattern was restricted to the bacteriocytes. Meanwhile, the scattered *Rickettsia* increased largely during the 21 days of post-adult emergence. The localization of *Wolbachia* within its arthropod hosts has been intensively studied, especially in *Drosophila* reproductive organs (ovaries and testes), but in whitefly hosts, only one study from Gottlieb *et al*. [20] revealed that *Wolbachia* was detected mostly at the circumference of and inside the bacteriocytes in the invasive *B*. *tabaci* Q biotype. Here, we investigate the infection dynamics and localization of *Wolbachia* in its *B*. *tabaci* AsiaII7 host via PCR and fluorescence in situ hybridization (FISH) methods. We propose that *Wolbachia* can also have varied localization patterns in *B*. *tabaci* AsiaII7 host and these patterns may not relate to the developmental stages of its whitefly host.

## Materials and Methods

Whitefly *B*. *tabaci* AsiaII7 cryptic species (formerly Cv biotype), which is a serious pest of agriculture in South China [21], was used in the current study. The AsiaII7 *B*. *tabaci* was originally collected from hibiscus (*Hibiscus rosa-sinensis*) in 2013 at Yuancheng city (114°41'28"E, 23°44'13"N), Guangdong province of China. The population was firstly reared on the same plant species in separate greenhouses at South China Agricultural University (SCAU) with ambient temperature, photoperiod and humidity, and then a subcolony was reared under constant laboratory conditions (26.0±0.5°C, RH 70–80%, 14:10 L:D photoperiod; light intensity was approximately 3000Lux) for experimental use. The purities of both greenhouse and laboratory populations were monitored monthly by sequencing the mitochondrial COI DNA according to the methods described by Qiu et al. [21].

### Detection of *Wolbachia* in AsiaII7 whitefly

The presence of *Wolbachia* in AsiaII7 *B*. *tabaci* at different developmental stages was detected by PCR method. The 3^rd^ - 4^th^ instar nymphs, male and female adults of AsiaII7 *B*. *tabaci* were individually homogenized in lysis buffer, while 15–20 eggs were homogenized together due to the potential low titer of *Wolbachia* within them. Whitefly DNA samples were extracted as previously described by Ahmed *et al*. [22]. The special primers used for *Wolbachia* detection were the *Wolbachia* surface protein (*wsp*) primers from Braig *et al*. [23] (wsp-81F: 5'-TGGTCCAATAAGTGATGAAG AAAC-3', wsp-691R: 5'-AAAAATTAAACGCTACT CCA-3') and the 16S rDNA primer from Li *et al*. [24] (315f: 5'-GCATGAGTGAAGAAGGCC-3', 628R-5'-AGATAGACGCCTTCGCCA-3'). The PCR procedure for *wsp* and 16S rDNA genes was as follows: firstly pre-denaturation at 95°C for 3 min then followed by 35 cycles of 94°C for 35 sec, 55°C for 30 sec and 72°C for 30 sec, and finally a 10 min extension period at 72°C. All PCRs were performed in a 25μl reaction volume that included 2.5 mM MgCl_2_, 200 mM for each dNTPs, 1μM of each primer, and 1 unit DNA Taq polymerase (Invitrogen, Guangzhou, China). After amplification, 5μl of the PCR product was visualized on a 1% agarose gel containing GoldView colourant and then photographed. When bands with the expected size were visible in the gels, the remaining 20μl of PCR product was sent for sequencing. Each PCR detection included a positive (DNA of *Portiera aleyrodidarum*) and negative (ddH_2_O) control to identify the DNA quality. Between 25–30 individuals of nymphs, male and female adults were screened in this experiment.

### FISH visualization of *Wolbachia* in AsiaII7 Whitefly

Eggs, 3^rd^ instar nymphs, male and female adults of AsiaII7 *B*. *tabaci* (20 individuals for each stage) were randomly collected and placed in Carnoy’s fixative. FISH detections were performed with the symbiont-specific 16S rRNA of *Wolbachia* (W2-Cy3: 5’-CTTCTGTGAGTACCGTCATTATC-3’) and the method described by Gottlieb *et al*. [25]. Stained whitefly samples were mounted and viewed under a Nikon eclipse Ti-U FluoView inverted microscope, and a no-probe staining AsiaII7 whitefly specimen was used as a negative control in the FISH detection. The individual numbers of different *Wolbachia* localization patterns in the nymph and adult stages of AsiaII7 were recorded. Experiments were repeated 3 times, and the proportion of whitefly individuals with scattered and confined *Wolbachia* were finally calculated.

In order to investigate whether the location patterns of *Wolbachia* change or not during the development of AsiaII7 immatures, the egg, nymph and adult samples (3–5 samples for each stage) from the same parent whitefly were collected. *Wolbachia* was visualized by FISH using the same probe and methods stated above. In an additional experiment, 5 females and their eggs (F1 generation) were collected separately. The location patterns of *Wolbachia* in the mother females and their eggs (F1 generation) were also examined by FISH. The experiments were repeated three times.

### Data analysis

The mean percentages of different *Wolbachia* localization patterns in the egg, nymph and adult stages of whitefly were analyzed using Proc Means program (SAS 9.2), and the differences were compared using t-test (PRT program, SAS, 9.2) at a significance level α = 0.05.

## Results

### *Wolbachia* infection in AsiaII7 whitefly

**Fig 1:**
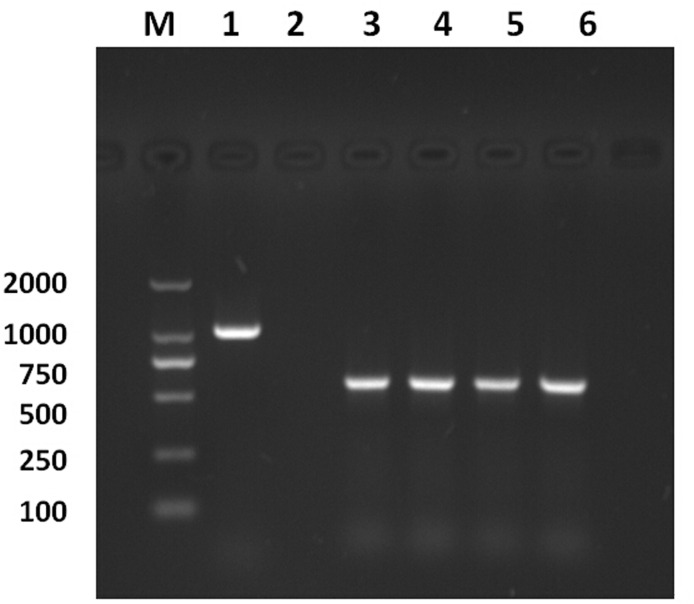
The infection of *Wolbachia* in the different stages of AsiaII7 *Bemisia tabaci*. *Wolbachia* was detected by PCR with the specific *wsp* primers, an expected DNA band of approximately 610 bp positively appeared in all the samples. M: DNA marker; Lanes 1–6 are positive control (*Portiera*, ~1000 bp), negative control (ddH_2_O), egg, nymph, male and female adults of AsiaII7 respectively.

The results of *Wolbachia* PCR screening based on *wsp* and 16S rDNA genes showed identical infection results both in the immature and adult stages of AsiaII7 *B*. *tabaci* including egg, nymph, male and female adults; all were infected with *Wolbachia* (Fig 1). The infection percentages of *Wolbachia* in nymph, male and female adults were 92.9% (26/28), 90.5% (19/21) and 96.2% (25/26), respectively, revealing a high infection status of *Wolbachia* in the AsiaII7 *B*. *tabaci* populations.

### FISH visualization of *Wolbachia* in AsiaII7 whitefly

**Fig 2:**
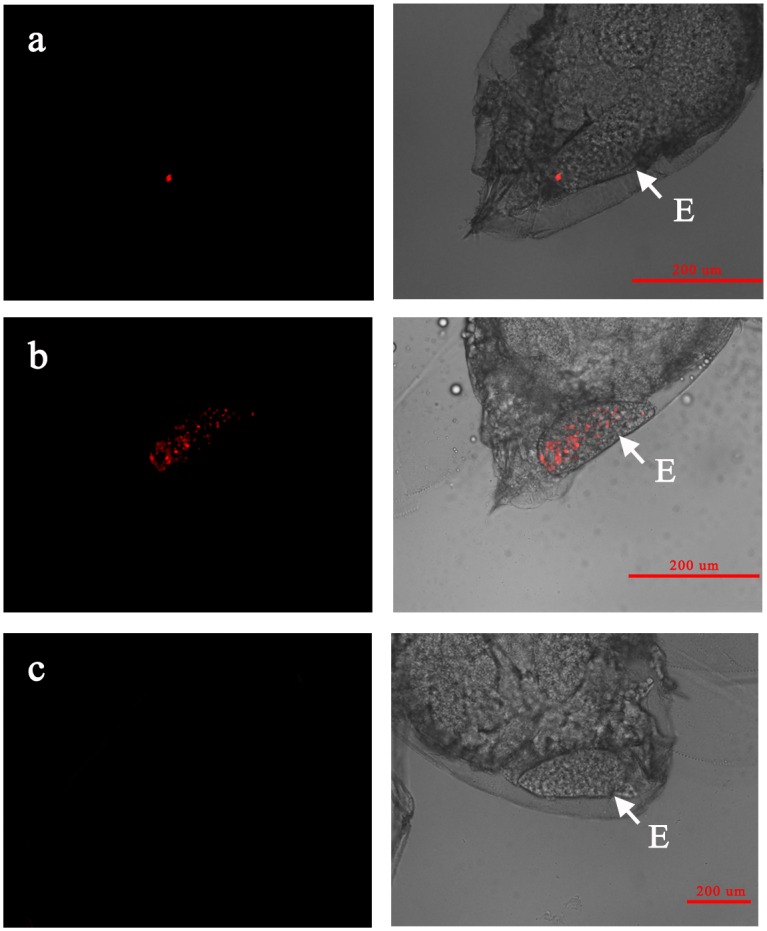
FISH visualization of *Wolbachia* in the eggs of AsiaII7 *Bemisia tabaci*. Panel a-b: confined and scattered *Wolbachia* in whitefly eggs; panel c: the negative control whitefly egg hybridization without specific probe. Left panels: fluorescence in dark field; right panels: fluorescence in bright field. E: AsiaII7 *B*. *tabaci* egg.

Results of fluorescence in situ hybridization revealed two localization patterns of *Wolbachia* in all the developmental stages of AsiaII7 *B*. *tabaci*, a scattered pattern and a confined pattern. In the eggs, the confined pattern of *Wolbachia* was very distinct, which was restricted in the bacteriome localized at one end of the whitefly egg (Fig 2a), while in the scattered pattern this symbiont was pervasive in the whole egg (Fig 2b). Moreover, it seems that more *Wolbachia* was concentrated in the pedicel end of the egg than other parts in the scattered localization pattern.

**Fig 3:**
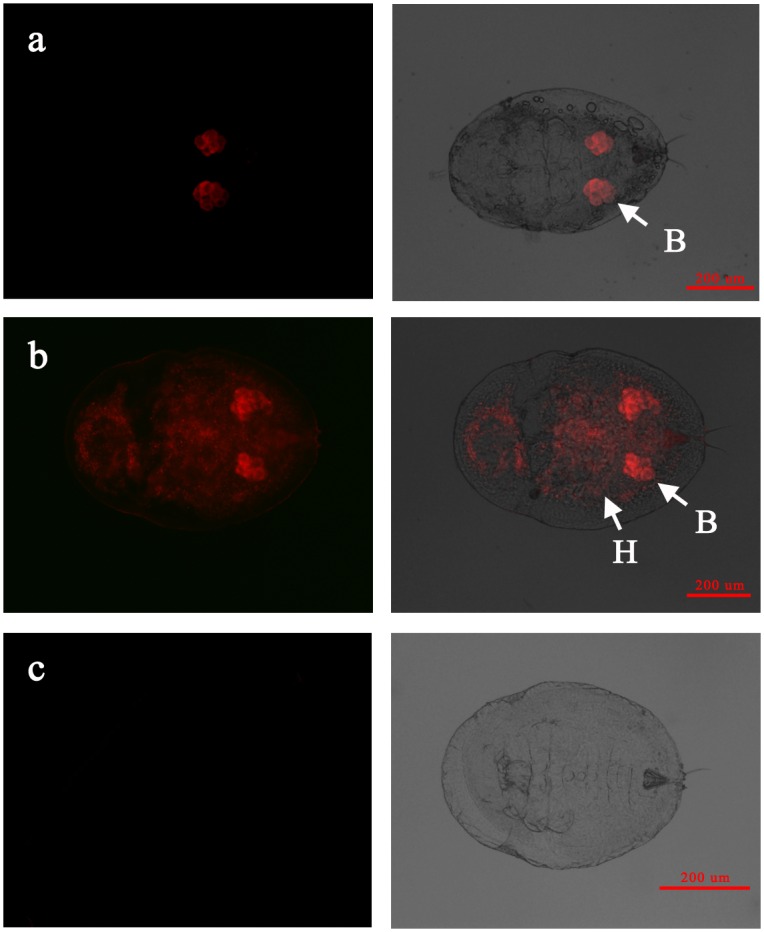
FISH visualization of *Wolbachia* in the nymphs of AsiaII7 *Bemisia tabaci*. Panel a-b: confined and scattered *Wolbachia* in nymphs; panel c: the negative control whitefly nymph hybridization without specific probe. Left panels: fluorescence in dark field; right panels: fluorescence in bright field. B: bacteriome in whitefly host, H: haemolymph tissue of whitefly host.

**Fig 4:**
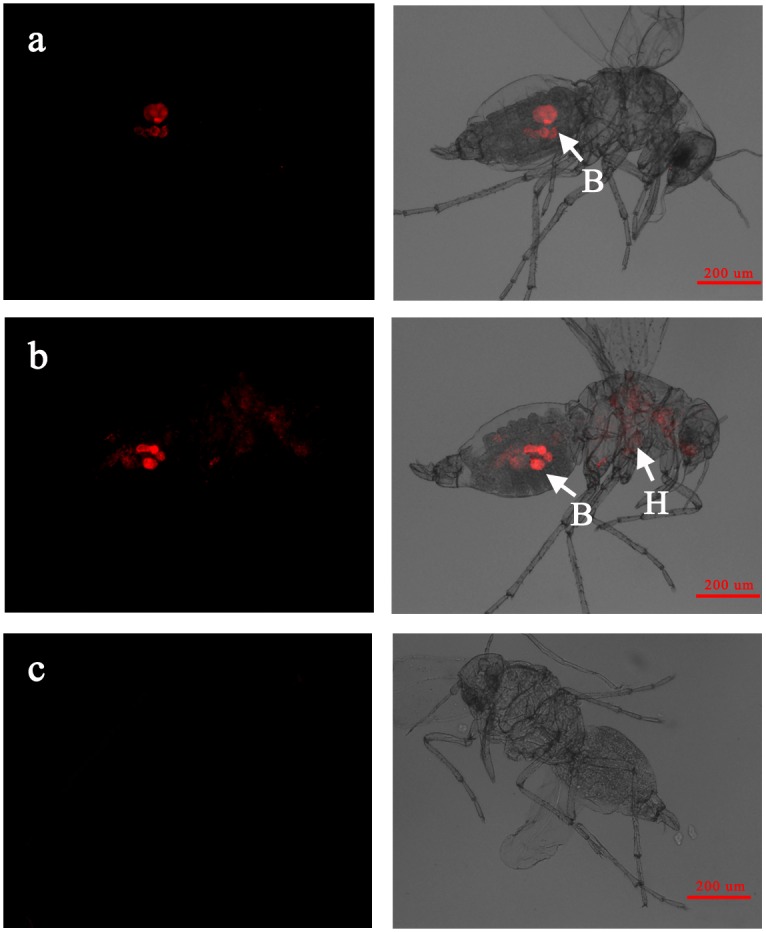
FISH visualization of *Wolbachia* in the male adults of AsiaII7 *B*. *tabaci*. Panel a-b: confined and scattered *Wolbachia* in male adults; panel c: the negative control whitefly male hybridization without specific probe. Left panels: fluorescence in dark field; right panels: fluorescence in bright field. B: bacteriome in whitefly host, H: haemolymph tissue of whitefly host.

**Fig 5:**
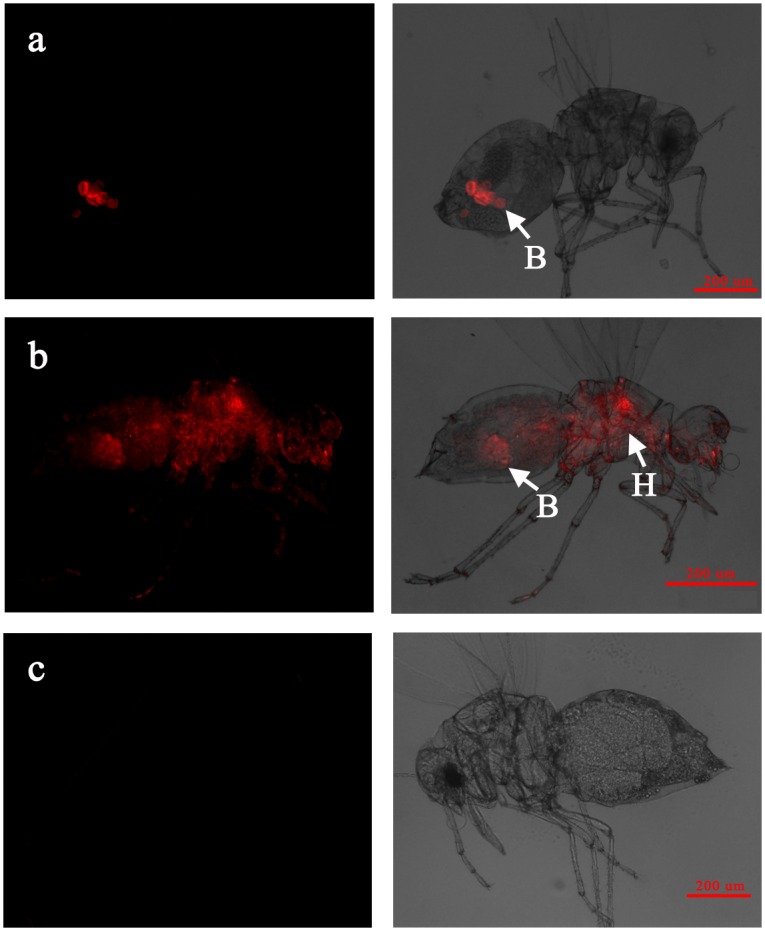
FISH visualization of *Wolbachia* in the female adults of AsiaII7 *B*. *tabaci*. Panel a-b: confined and scattered *Wolbachia* in female adults; panel c: the negative control whitefly female hybridization without specific probe Left panels: fluorescence in dark field; right panels: fluorescence in bright field. B: bacteriome in whitefly host, H: haemolymph tissue of whitefly host.

When infecting the AsiaII7 whitefly nymph in a confined pattern, *Wolbachia* was detected mostly around or inside the bacteriomes located in the abdomen of hosts (Fig 3a). However, on the contrary, the scattered *Wolbachia* symbiont was visualized not only in the bacteriomes but also in different body regions of nymph (Fig 3b). The localization of *Wolbachia* in the adults of AsiaII7 *B*. *tabaci* was similar to that in the nymphs; confined symbiont was restricted to the bacteriomes in the abdomens of males and females, and scattered *Wolbachia* was found both in and outside of the bacteriomes, located in the organs of abdomen, thorax and head (Figs 4a and 4b, 5a and 5b). The detection of the location patterns of *Wolbachia* in the egg, nymph and adult samples from same parent whitefly, indicated that location patterns did not change during the whole developmental period. Adult females infected with confined *Wolbachia* always produced *Wolbachia*-confined eggs, while those infected with scattered *Wolbachia* always produced *Wolbachia*-scattered eggs. In addition, our results also indicated that the distribution patterns of *Wolbachia* symbiont were not associated with the sex of whitefly host.

### Dynamics of *Wolbachia* localization in AsiaII7 whitefly

**Fig 6:**
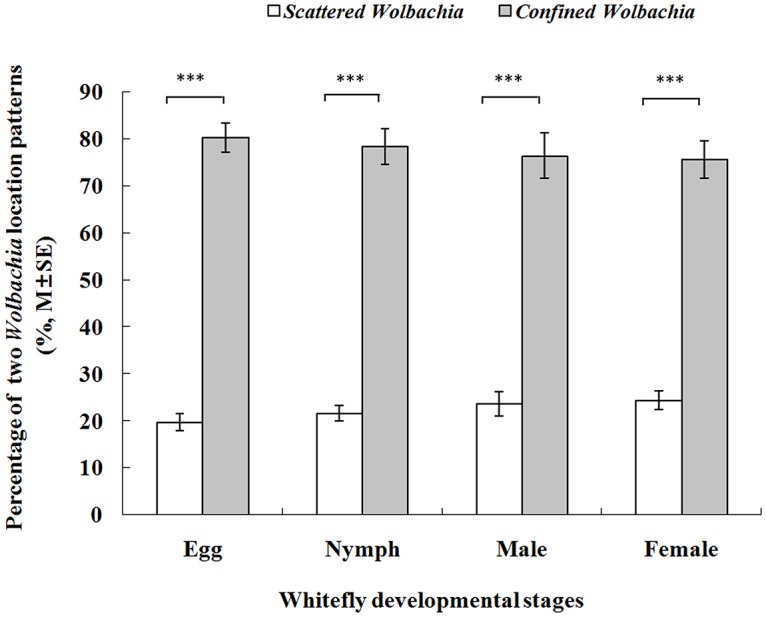
The percentage of *Wolbachia* location patterns in different stages of AsiaII7 *Bemisia tabaci*. For egg, nymph, male and female adults, 56, 46, 47 and 57 individuals (three repeats in total) were successfully visualized by FISH respectively. The differences between the percentages of scattered and confined *Wolbachia* were compared using t-test, the “***” over the bars mean significantly differences between the percentages at α = 0.01 level. T = -24.30, P = 0.0017 for egg; T = -27.89, P = 0.0013 for nymph; T = -16.09, P = 0.0038 for male and T = -22.02, P = 0.0021 for female adults.

Although both the confined and scattered localization patterns of *Wolbachia* were detected in the AsiaII7 *B*. *tabaci*, FISH visualization results revealed that the scattered localization pattern was significantly lower than the confined one. The percentages of host individuals infected with scattered *Wolbachia* were 19.73 ±1.85%, 21.6 ±1.62%, 23.6±2.64% and 24.4±1.96% for egg nymph, adult males and adult females, respectively (Fig 6, S1 Table, M±SE). Again, it seems that the distribution patterns of *Wolbachia* symbiont are not associated to the developmental stage (nymph or adult) and sex of host (male or female).

## Discussion

During the past two decades, the associations of bacterial endosymbionts and their arthropod hosts have become a matter of interest. A significant increase in studies on related topics suggests that these symbionts play significant roles in the biology of their hosts [8, 26–28]. Among arthropods, sap-feeding insects such as whitefly, aphid, psyllid and leafhopper, usually harbor abundant species of secondary endosymbionts, including *Rickettsia*, *Wolbachia*, *Arsenophonus*, *Cardinium*, *Serratia* and *Regiella* [20, 29–33]. Endosymbionts are usually diverse in different host species, and even or in different geographical populations of the same host species. For example, *B*. *tabaci* is a small hemipterous insect that feeds on the phloem sap of numerous host plants. It is currently considered as a complex of at least 24 distinct cryptic species, which are morphologically indistinguishable but differ markedly in their host range, ability to transmit viruses and the endosymbionts they are infected with [28, 34]. Therefore, investigation on individual endosymbiont species and their distribution patterns has been an area of interest due to their important effects on host fitness. In this study, although the confocal microscope images of primary *Portiera* and secondary *Wolbachia* are not available, we have systematically and distinctly showed two different localization patterns of *Wolbachia* in all the developmental stages of the same whitefly host, AsiaII7 *B*. *tabaci*. Moreover, the distribution patterns were similar to those previously found in *Rickettsia* symbiont. The differences in symbiont localization were thought to be the results of a genetic modification in host factors that control the movement of symbiont, or of a change in the bacterium itself so affecting mobility [3].

It is well known that the obligate primary endosymbionts such as *Buchnera* in aphids, *Portiera* in whiteflies and *Carsonella* in psyllids are generally confined and localized in the special cell bacteriocytes, which are highly related to strictly vertical transmission from parents to offspring [3, 29, 35], while the secondary symbionts are generally distributed in other organs of host insects. Thus, it is speculated that the various locations of these symbionts may be highly associated with their different physiological roles in their hosts [6, 8, 36–38]. Primary symbionts are essential for host survival and development, providing the host with essential amino acids and vitamins [3]. Thus, the long term co-evolution of primary symbionts with an insect host has meant the forming of their own mechanisms to ensure their vertical transmission. For secondary symbionts, their distributions may indicate that they are not necessary for host survival, but they may have more functions relating to host biology, so affecting the phenotype of the host. In this study, the scattered *Wolbachia* was found both in and outside of the bacteriomes, distributed in the reproductive system and other tissues of AsiaII7 *B*. *tabaci* host, whereas the confined *Wolbachia* was only found within the bacteriomes. The DNA sequencing of 16S rDNA and *wsp* genes revealed that these two patterns of *Wolbachia* are 100% and 99.9% identical to each other (BLQ unpublished data), but whether their physiological functions are same or not, still needs to be further investigated.

*Wolbachia* is not the only endosymbiont with more than one localization pattern in its whitefly host. In *B*. *tabaci* Middle East-Asia Minor 1 cryptic species (MEAM1, formerly B biotype), Caspi-Fluger et al. [3] revealed that the secondary symbiont *Rickettsia* presents two distinct localization patterns throughout development and adulthood in its whitefly host, which is similar to the *Wolbachia* location patterns discovered in current study: in the scattered pattern, *Rickettsia* is localized throughout the whitefly hemocoel, excluding the bacteriocytes, while in the confined pattern, *Rickettsia* is restricted to the bacteriocytes. In pea aphids, *Rickettsia*, *Hamiltonella*, *Serratia* and *Regiella* were found in three localizations within their hosts: secondary bacteriocytes, oocytes, sheath cells, salivary glands and haemolymph [31, 39, 40]. The localizations of these symbionts in these organs may give us some clues to their possible horizontal transmission routes. For example, by localizing in the reproductive system, male-borne symbionts can be acquired by females and subsequently established stable, maternally transmitted associations [41], by localizing in the salivary glands, *Rickettsia* can be inputted into the phloem of a plant by a donor whitefly and then easily taken up by a recipient whitefly feeding on the same plant leaves [42]. In addition, when localized in the haemolymph, *Wolbachia* has a high possibility to be phoretically picked up by parasitoids when they are probing to check a donor whitefly nymph and therefore input this symbiont into another individual during the next probing exercise [43].

In conclusion, during this study we have shown that one insect host can harbor different distribution patterns of *Wolbachia* in the bacteriocytes and haemolymph simultaneously. These new findings are helpful to understand why there is a high abundance of symbionts including *Wolbachia* in arthropod communities in nature. The physiological roles of these symbionts in different localization patterns should be further investigated, as most of them are directly involved in phenotype characteristics of their individual host species, including virus transmission, chemical resistance, heat tolerance, host’s immunity and also host protection against parasites and pathogens.

## Supporting Information

S1 Table The related raw experimental data for Fig 6, Location patterns of *Wolbachia* in the different stages of AsiaII7 *B*. *tabaci*. (XLS)

## References

[1] Perlman SJ, Hunter MS, Zchori-Fein E. The emerging diversity of *Rickettsia*. P R Soc B. 2006;273(1598):2097–106. WOS:000240104300001. 10.1098/rspb.2006.3541 PMC1635513 16901827

[2] Watts T, Haselkorn TS, Moran NA, Markow TA. Variable incidence of *Spiroplasma* infections in natural populations of *Drosophila* species. PLoS One. 2009;4(5). WOS:000266415100007. 10.1371/journal.pone.0005703 PMC2683927 19492088

[3] Caspi-Fluger A, Inbar M, Mozes-Daube N, Mouton L, Hunter MS, Zchori-Fein E. *Rickettsia* 'In' and 'Out': Two different localization patterns of a bacterial symbiont in the same insect species. PLoS One. 2011;6(6). WOS:000291985100010. 10.1371/journal.pone.0021096 PMC3119683 21712994

[4] Moran NA, McCutcheon JP, Nakabachi A. Genomics and evolution of heritable bacterial symbionts. Annu Rev Genet. 2008;42:165–90. 10.1146/annurev.genet.41.110306.130119. WOS:000261767000008. 18983256

[5] Hedges LM, Brownlie JC, O'Neill SL, Johnson KN. *Wolbachia* and virus protection in insects. Science. 2008;322(5902):702-. WOS:000260605200037. 10.1126/science.1162418 18974344

[6] Montllor CB, Maxmen A, Purcell AH. Facultative bacterial endosymbionts benefit pea aphids *Acyrthosiphon pisum* under heat stress. Ecol Entomol. 2002;27(2):189–95.

[7] Oliver KM, Moran NA, Hunter MS. Variation in resistance to parasitism in aphids is due to symbionts not host genotype. P Natl Acad Sci USA. 2005;102(36):12795–800. 10.1073/pnas.0506131102. WOS:000231716700029. PMC1200300 16120675

[8] Oliver KM, Russell JA, Moran NA, Hunter MS. Facultative bacterial symbionts in aphids confer resistance to parasitic wasps. P Natl Acad Sci USA. 2003;100(4):1803–7. 10.1073/pnas.0335320100. WOS:000181073000066. PMC149914 12563031

[9] Stouthamer R, Breeuwer JA, Hurst GD. *Wolbachia pipientis*: microbial manipulator of arthropod reproduction. Annu Rev Microbiol. 1999;53:71–102. 10547686 10.1146/annurev.micro.53.1.71

[10] Dobson SL, Bourtzis K, Braig HR, Jones BF, Zhou W, Rousset F, et al *Wolbachia* infections are distributed throughout insect somatic and germ line tissues. Insect Biochem Molec. 1999;29(2):153–60. 10.1016/s0965-1748(98)00119-2. BIOSIS:PREV199900218641. 10196738

[11] Min KT, Benzer S. *Wolbachia*, normally a symbiont of *Drosophila*, can be virulent, causing degeneration and early death. P Natl Acad Sci USA. 1997;94(20):10792–6. 10.1073/pnas.94.20.10792 PMC23488 9380712

[12] Zchori-Fein E, Roush RT, Rosen D. Distribution of parthenogenesis-inducing symbionts in ovaries and eggs of *Aphytis* (Hymenoptera: Aphelinidae). Curr Microbiol. 1998;36(1):1–8. 9405738 10.1007/s002849900270

[13] Ijichi N, Kondo N, Matsumoto R, Shimada M, Ishikawa H, Fukatsu T. Internal spatiotemporal population dynamics of infection with three *Wolbachia* strains in the adzuki bean beetle, *Callosobruchus chinensis* (Coleoptera: Bruchidae). Appl Environ Microb. 2002;68(8):4074–80. WOS:000177260500052. 10.1128/AEM.68.8.4074-4080.2002 PMC124025 12147509

[14] Mitsuhashi W, Saiki T, Wei W, Kawakita H, Sato M. Two novel strains of *Wolbachia* coexisting in both species of mulberry leafhoppers. Insect Mol Biol. 2002;11(6):577–84. WOS:000179068300007. 12421415 10.1046/j.1365-2583.2002.00368.x

[15] Werren JH. Biology of *Wolbachia*. Annu Rev Entomol. 1997;42:587–609. 15012323 10.1146/annurev.ento.42.1.587

[16] Zug R, Hammerstein P. Still a host of hosts for *Wolbachia*: Analysis of recent data suggests that 40% of terrestrial arthropod species are infected. PLoS One. 2012;7(6). WOS:000305351700044. 10.1371/journal.pone.0038544 PMC3369835 22685581

[17] Weinert LA, Araujo-Jnr EV, Ahmed MZ, Welch JJ. The incidence of bacterial endosymbionts in terrestrial arthropods. P R Soc B. 2015;282(1807). 10.1098/Rspb.2015.0249. WOS:000353351100009. PMC4424649 25904667

[18] Voronin D, Tran-Van V, Potier P, Mavingui P. Transinfection and growth discrepancy of *Drosophila Wolbachia* strain wMel in cell lines of the mosquito *Aedes albopictus*. J Appl Microbiol. 2010;108(6):2133–41. WOS:000277412600028. 10.1111/j.1365-2672.2009.04621.x 19951376

[19] Werren JH, Baldo L, Clark ME. *Wolbachia*: master manipulators of invertebrate biology. Nat Rev Microbiol. 2008;6(10):741–51. WOS:000259217200022.. 10.1038/nrmicro1969 18794912

[20] Gottlieb Y, Ghanim M, Gueguen G, Kontsedalov S, Vavre F, Fleury F, et al Inherited intracellular ecosystem: symbiotic bacteria share bacteriocytes in whiteflies. Faseb J. 2008;22(7):2591–9. 10.1096/Fj.07-101162. WOS:000257292500050. 18285399

[21] Qiu BL, Chen YP, Liu L, Peng WL, Li XX, Ahmed MZ, et al Identification of three major *Bemisia tabaci* biotypes in China based on morphological and DNA polymorphisms. Prog Nat Sci. 2009;19(6):713–8. 10.1016/j.pnsc.2008.08.013. WOS:000266344900008.

[22] Ahmed MZ, Ren SX, Xue X, Li XX, Jin GH, Qiu BL. Prevalence of endosymbionts in *Bemisia tabaci* populations and their in vivo sensitivity to antibiotics. Curr Microbiol. 2010;61(4):322–8. 10.1007/s00284-010-9614-5. WOS:000281978900014. 20217091

[23] Braig HR, Zhou W, Dobson SL, O'Neill SL. Cloning and characterization of a gene encoding the major surface protein of the bacterial endosymbiont *Wolbachia pipientis*. J Bacteriol. 1998;180(9):2373–8. 9573188 10.1128/jb.180.9.2373-2378.1998 PMC107178

[24] Li ZX, Lin HZ, Guo XP. Prevalence of *Wolbachia* infection in *Bemisia tabaci*. Curr Microbiol. 2007;54(6):467–71. WOS:000246618100013. 17487529 10.1007/s00284-007-0011-7

[25] Gottlieb Y, Ghanim M, Chiel E, Gerling D, Portnoy V, Steinberg S, et al Identification and localization of a *Rickettsia* sp in *Bemisia tabaci* (Homoptera: Aleyrodidae). Appl Environ Microb. 2006;72(5):3646–52. WOS:000237491200069. 10.1128/AEM.72.5.3646-3652.2006 PMC1472322 16672513

[26] Siozios S, Sapountzis P, Ioannidis P, Bourtzis K. *Wolbachia* symbiosis and insect immune response. Insect Sci. 2008;15(1):89–100. WOS:000252587700007.

[27] Brumin M, Kontsedalov S, Ghanim M. *Rickettsia* influences thermotolerance in the whitefly *Bemisia tabaci* B biotype. Insect Sci. 2011;18(1):57–66. WOS:000286476000007.

[28] Su Q, Oliver KM, Pan HP, Jiao XG, Liu BM, Xie W, et al Facultative symbiont *Hamiltonella* confers benefits to *Bemisia tabaci* (Hemiptera: Aleyrodidae), an invasive agricultural pest worldwide. Environ Entomol. 2013;42(6):1265–71. 10.1603/En13182. WOS:000329990000016. 24280594

[29] Baumann P. Biology of bacteriocyte-associated endosymbionts of plant sap-sucking insects. Annu Rev Microbiol. 2005;59:155–89. 10.1146/annurev.micro.59.030804.121041. WOS:000233054800009. 16153167

[30] Chiel E, Gottlieb Y, Zchori-Fein E, Mozes-Daube N, Katzir N, Inbar M, et al Biotype-dependent secondary symbiont communities in sympatric populations of *Bemisia tabaci*. B Entomol Res. 2007;97(4):407–13. 10.1017/S0007485307005159. WOS:000249014700009. 17645822

[31] Fukatsu T, Nikoh N, Kawai R, Koga R. The secondary endosymbiotic bacterium of the pea aphid *Acyrthosiphon pisum* (Insecta: Homoptera). Appl Environ Microb. 2000;66(7):2748–58. 10.1128/aem.66.7.2748-2758.2000 PMC92069 10877764

[32] Zhang KJ, Han X, Hong XY. Various infection status and molecular evidence for horizontal transmission and recombination of *Wolbachia* and *Cardinium* among rice planthoppers and related species. Insect Sci. 2013;20(3):329–44. WOS:000318949600007. 10.1111/j.1744-7917.2012.01537.x 23955885

[33] Sacchi L, Genchi M, Clementi E, Bighardi E, Avanzati AM, Pajoro M, et al Multiple symbiosis in the leafhopper *Scaphoideus titanus* (Hemiptera: Cicadellidae): Details of transovarial transmission of *Cardinium* sp and yeast-like endosymbionts. Tissue Cell. 2008;40(4):231–42. 10.1016/j.tice.2007.12.005 18272191

[34] De Barro PJ, Liu SS, Boykin LM, Dinsdale AB. *Bemisia tabaci*: A statement of species status. Annl Rev Entomol. 2011;56:1–19. 10.1146/annurev-ento-112408-085504. WOS:000286841900001. 20690829

[35] Nakabachi A, Shigenobu S, Sakazume N, Shiraki T, Hayashizaki Y, Carninci P, et al Transcriptome analysis of the aphid bacteriocyte, the symbiotic host cell that harbors an endocellular mutualistic bacterium, *Buchnera*. P Natl Acad Sci USA. 2005;102(15):5477–82. WOS:000228376600035. 10.1073/pnas.0409034102 PMC555734 15800043

[36] Sakurai M, Koga R, Tsuchida T, Meng XY, Fukatsu T. *Rickettsia* symbiont in the pea aphid *Acyrthosiphon pisum*: Novel cellular tropism, effect on host fitness, and interaction with the essential symbiont *Buchnera*. Appl Environ Microb. 2005;71(7):4069–75. WOS:000230445700083. 10.1128/AEM.71.7.4069-4075.2005 PMC1168972 16000822

[37] Tsuchida T, Koga R, Fukatsu T. Host plant specialization governed by facultative symbiont. Science. 2004;303(5666):1989 10.1126/science.1094611. WOS:000220429800032. 15044797

[38] Ferrari J, Darby AC, Daniell TJ, Godfray HCJ, Douglas AE. Linking the bacterial community in pea aphids with host-plant use and natural enemy resistance. Ecol Entomol. 2004;29(1):60–5.

[39] Koga R, Tsuchida T, Fukatsu T. Changing partners in an obligate symbiosis: a facultative endosymbiont can compensate for loss of the essential endosymbiont *Buchnera* in an aphid. P R Soc B. 2003;270(1533):2543–50. 10.1098/rspb.2003.2537. WOS:000187989900002. PMC1691542 14728775

[40] Tsuchida T, Koga R, Meng XY, Matsumoto T, Fukatsu T. Characterization of a facultative endosymbiotic bacterium of the pea aphid *Acyrthosiphon pisum*. Microb Ecol. 2005;49(1):126–33. 10.1007/s00248-004-0216-2. WOS:000228012700012. 15690225

[41] Moran NA, Dunbar HE. Sexual acquisition of beneficial symbionts in aphids. P Natl Acad Sci USA. 2006;103(34):12803–6. 10.1073/pnas.0605772103. WOS:000240035900030. PMC1568928 16908834

[42] Caspi-Fluger A, Inbar M, Mozes-Daube N, Katzir N, Portnoy V, Belausov E, et al Horizontal transmission of the insect symbiont *Rickettsia* is plant-mediated. P R Soc B. 2012;279(1734):1791–6. WOS:000301981300017. 10.1098/rspb.2011.2095 PMC3297456 22113034

[43] Ahmed MZ, Li SJ, Xue X, Yin XJ, Ren SX, Jiggins FM, et al The intracellular bacterium *Wolbachia* uses parasitoid wasps as phoretic vectors for efficient horizontal transmission. PLoS Pathog. 2015;10(2):e1004672 10.1371/journal.ppat.1004672 25675099 PMC4347858

